# Regulating the Balance of Th17/Treg via Electroacupuncture and Moxibustion: An Ulcerative Colitis Mice Model Based Study

**DOI:** 10.1155/2017/7296353

**Published:** 2017-12-17

**Authors:** Jungang Sun, Hejiaozi Zhang, Chengyulin Wang, Mingxiao Yang, Shyang Chang, Yu Geng, Hui Yang, Zhiqi Zhuang, Xiang Wang, Lushuang Xie, Biao Huang, Na Zhao, Wei Zhou, Xinhui Cheng, Bei Cai, Qiaofeng Wu, Shu Guang Yu

**Affiliations:** ^1^Acupuncture and Tuina College, Chengdu University of Traditional Chinese Medicine, Chengdu, Sichuan 610075, China; ^2^Sichuan Integrative Medicine Hospital, 4th Ren Ming Road, Sichuan 610041, China; ^3^The First Affiliated Hospital of Chengdu Medical College, 278 Baoguang Avenue, Xindu District, Chengdu, Sichuan 610500, China; ^4^Department of Electrical Engineering, National Tsing Hua University, Hsinchu 300, Taiwan; ^5^Dazhou Integrative Medicine Hospital, Dazhou, Sichuan 635000, China; ^6^West China Hospital, Sichuan University, Chengdu, Sichuan 610014, China

## Abstract

**Aim:**

To investigate the relationship between the effects of electroacupuncture/moxibustion and the balance of Th17/Treg in treating ulcerative colitis (UC) and to preliminary compare the effects of the above two methods.

**Methods:**

DSS-induced UC mice were treated by electroacupuncture and moxibustion. Disease activity index (DAI) was scored; intestinal pathological structure and ultrastructure were observed. The levels of IL-2, IL-6, IL-10, IL-17A, IL-17F, and TGF-*β* in plasma were measured by ELISA. The percentages of Treg and Th17 in spleen lymphocytes were analyzed by flow cytometry. Also, the expressions of TLR2, TLR4, ROR*γ*t, and FOXP3 in the distal colon were detected by immunohistochemistry or western blot.

**Results:**

Both electroacupuncture and moxibustion can relieve UC. These effects are further supported by ELISA results. In addition, the ratio of Treg and Th17 in spleen lymphocytes and the expression of TLR2 and TLR4 are significantly improved. Also, the expression of ROR*γ*t and FOXP3 in distal colon were improved. Besides, the effect of moxibustion is better than that of electroacupuncture on TLR2, TLR4, and FOXP3 expression (*P* < 0.05).

**Conclusion:**

Both electroacupuncture and moxibustion may ameliorate UC by regulating the balance of Th17/Treg. Whether moxibustion has better efficacy than electroacupuncture needs further study.

## 1. Introduction

Ulcerative colitis (UC) is a common kind of chronic colonic inflammation disease in north America and northern Europe with incidence varying from nine to twenty cases per one hundred thousand person-years [1]. A population-based cohort study proved that despite an overall normal life expectancy for patients with ulcerative colitis, patients >50 years of age and with extensive colitis at diagnosis had increased mortality within the first 2 years after diagnosis. Therefore, it is very important to control the disease progression [2]. In addition, with the increasing adoption of industrialized lifestyle, the incidence of UC has increased in developing country such as China [3]. Therefore, the management of UC is very critical worldwide.

The primary symptoms of UC are abdominal pain, diarrhea, bloody stool, and weight loss [4, 5]. The causes that can induce UC are many, such as genetic, infection, immunity, and environment to name a few. While previous studies have shown that Th1/Th2 may play a crucial role in the production of UC [6], recent studies however have found that CD4^+^ T cell subsets Treg and Th17 form a new immune axis and are closely related to the occurrence and development of UC [6–8]. First of all, Treg cells orchestrate the overall immune response and play important roles in preventing the aggravation of UC by regulating the activity of effect T cells. It suppresses the activity of effect cells by secreting TGF-*β*1 and/or IL-10 which inhibit the proliferation of Th cells and their production of inflammatory factor [9]. On the other hand, Th17 cells, as a key effecter in the immune response, play critical roles in the development of autoimmunity by producing IL-17 and other proinflammatory cytokines such as IL-6, IL-21, IL-22, and TNF-*α*, which in turn induce the migration of neutrophils towards sites of infection to elicit an inflammatory response [7, 10, 11]. Many studies, so far, have shown that these two kinds of cells form an immune balance as a switch of a variety of immune diseases including UC [12].

Conventional drugs for UC include aminosalicylates, corticosteroids, antibiotics, and immunomodulators [13]. However, side effects such as nausea, vomiting, headaches, rash, fever, agranulocytosis, pancreatitis, nephritis, hepatitis, and male infertility have been widely reported using these drugs [14]. As a result, complementary and alternative medicine may play an important role in dealing with UC without causing any one of the aforementioned side effects [15]. For instance, many patients, especially in China and Asia areas, try to consult traditional medical doctors to manage their conditions by acupuncture and moxibustion [16]. Up till now, reports have demonstrated that acupuncture plus moxibustion or moxibustion alone can (1) manage the symptoms of abdominal pain, diarrhea, bloody stool, and consequent weight loss [17], (2) promote recovery of damaged intestinal tissues due to inflammation [18], and (3) suppress the inflammatory response of UC [19]. However, as to the Th17/Treg, so far very few studies of acupuncture and moxibustion have direct evidence to show that they can influence the balance of these two kinds of cells. What is more, study which directly compared the effect of acupuncture with that of moxibustion for treating UC is lacking. Therefore, in this study, we will design the animal study so as to attest if acupuncture and moxibustion can ameliorate UC and whether their effects are obtained from improving the balance of Th17/Treg. In addition, we will also compare the effects of acupuncture and moxibustion to see which one is better in alleviating UC based on animal experiment.

## 2. Materials and Methods

### 2.1. Ethics Statement

All experimental animals were purchased from Laboratory Animal Services Center of Sichuan Provincial People's Hospital (Sichuan, China). All experimental procedures were approved by the Animal Care and Use Committee of Chengdu University of Traditional Chinese Medicine.

### 2.2. Animals and UC Model Inducing

Male Kun Ming mice (36 ± 2 g, 6–8 weeks) were housed in an environmentally controlled vivarium under a 12 h light–dark cycle (temperature 20 ± 2°C, humidity 50–60%). Food and water were available ad libitum. After 1 week's adaptation, the mice were divided into 4 groups randomly; among them, three groups were induced to ulcerative colitis model by drinking 3% Dextran Sodium Sulfate (DSS, 43 kDa, MP Biomedicals) [20]. The control group received ordinary water only. After successful setting of the model, the UC model mice were randomly divided into UC model group, electroacupuncture group, and moxibustion group.

### 2.3. Electroacupuncture and Moxibustion Treatment

From the 5th day of modeling, treatments were performed. In the electroacupuncture group, acupoints “Guanyuan” (RN4) and “Zusanli” (ST36) were considered to be effective in treating UC according to clinic practice [21] and our previous studies [22, 23]. Acupoints “Guanyuan” (RN4) and “Zusanli” (ST36) were chosen for their effectiveness in improving the symptoms of UC human patients [21–23]. The right and left acupoints of ST36 will be used alternately. The locations for these acupoints were determined according to Government Channel and Points Standard GB12346-90 of China and “The Veterinary Acupuncture of China.” One-channel electrical stimulations were performed via the two stainless-steel acupuncture needles (Ø 0.25 × 0.25 mm) being inserted into the acupoints with a pulse generator (Model 1002378080; China Medical Supplies Factory Co., Ltd., Suzhou, China). The electrical stimuli consisted of pulse trains with a frequency range from 2 to 100 Hz with dilatational wave and voltage range from 2 to 4 V. The current is 0.1 mA. The moxa stick (diameter 0.5 cm, length 15 cm) was ignited and hung 1.5 cm above acupoints RN4 and ST36. Each acupoint was used in the treatment for 10 min every day. The whole treatment course comprised 5 days.

### 2.4. Sample Collection

Body weight was measured every day. Occult blood tests were used to evaluate the UC symptom. After five days of treatment, all animals were anesthetized with 1% pentobarbital sodium at a concentration of 3 ml/kg. One-milliliter blood samples were collected by eyeball removal into a heparin tube. Spleens were removed, from which lymphocytes were collected and centrifuged, and single cell suspensions of splenocytes were stimulated and detected by flow cytometry. The distal colon tissues from mice were either embedded in paraffin blocks and sectioned, or fixed in formaldehyde, or frozen in liquid nitrogen and subsequently stored at −80°C for later western blot test.

### 2.5. Disease Activity Evaluation of UC Mice

The mice were observed daily for morbidity and given a clinical disease score (disease activity index, DAI) between 0 and 15 based on the following characteristic criteria: body weight loss, characteristics of feces, and fecal occult blood (Table 1). The DAI is expressed as the equation: DAI = (body weight loss + characteristics of feces + fecal occult blood)/3 [24].

### 2.6. Histological Observation

The distal colonic tissue (2 cm beyond the anus, the total segment length being about 4 cm) was fixed and paraffin-embedded and stained with hematoxylin and eosin (H&E) to allow histological examination. We further observed the morphology of distal colonic mucosal epithelia by electron microscopy.

### 2.7. ELISA Assay

The levels of IL-6, IL-10, IL-17A, IL-17F, and transforming growth factor *β* (TGF-*β*) in blood were detected by typical enzyme-linked immunosorbent assay (ELISA) with commercially available kits (Shanghai Blue Gene Biotech Co., Ltd., China), according to the manufacturer's protocol.

### 2.8. Immunohistochemical Staining and Semiquantitative Analysis

To evaluate the serious degree of inflammation, the expressions of the toll-like receptors (TLR2 and TLR4, bought from Abcam Inc., USA) were detected in the colon tissue by the immunohistochemical method. Briefly, colonic tissues were fixed in 4.0% buffered paraformaldehyde and paraffin-embedded. 5 *μ*m thick sections were taken from the paraffin blocks and the immunohistochemical studies were performed on this section. 3.0% bovine serum albumin (BSA) was used for 1 h to reduce nonspecific antibody binding. The slides were incubated overnight at 4°C with mouse TLR2 and TLR4 antibodies (dilution 1 : 200). Secondary antibody and streptavidin biotinylated tests were performed later. All the immunohistochemical slides were semiquantitatively analyzed and interpreted by two researchers blinded to the treatment. Five random fields at 400x magnification were counted in each sectioned sample. The relative optical density was quantified by the Image-Pro Plus 6.0 (Media Cybernetics, USA).

### 2.9. Flow Cytometry

The spleen lymphocytes were collected and centrifuged, and single cell suspensions of splenocytes were stimulated. For Treg analysis, FITC (BD Biosciences, USA), PE (BD Biosciences, USA), and Foxp3 PerCP-cy5.5 (eBioscience, USA) were used for dying CD4+CD25+Foxp3+Treg cells and their corresponding isotype controls. For Th17 analysis, lymphocytes from spleen of each group were stimulated with ionomycin, PMA, and monensin for 5 h. FITC, PE, and IL-17A PerCP-cy5.5 (eBioscience, USA) were used for dying CD3+CD8+IL-17+Th17 cells and their respective isotype controls (from BD Biosciences, USA, and eBioscience, USA, resp.). Then, the single cell suspensions of splenocytes were incubated and washed. The levels of Treg and Th17 cytokines in the spleen lymphocytes were detected by flow cytometry (FC-500, Beck Coulter, USA).

### 2.10. Western Blot

Protein extracts were prepared from the distal colon tissues using a lysis buffer supplemented with ethylenediaminetetraacetic acid- (EDTA-) free complete protease inhibitors. Proteins were extracted and subjected to sodium dodecyl sulfate polyacrylamide gel electrophoresis (SDS-PAGE) on a 10% gel. The bands were transferred to a polyvinylidene difluoride membrane. After blocking with 5% nonfat dry milk in Tris-buffered saline supplemented with 0.1% Tween 20 for 4 h at room temperature, the membranes were incubated overnight at 4°C with the following antibodies: TLR2, TLR4, and ROR*γ*t (Abcam Inc., USA) and FOXP3 (Abcam Inc., USA) primary antibodies (all used at 1 : 1000 dilution). The membranes were then incubated with a secondary antibody at 37°C for 1 h. Normalization was performed by blotting the same membranes with anti-*β*-actin antibody (Abcam Inc., USA).

### 2.11. Statistical Analysis

All data were presented as the mean ± SD and evaluated by one-way ANOVA performed using the software SPSS version 12.0 (SPSS Inc., Chicago, IL, USA). For *P* < 0.05, it will be regarded as statistically significant.

## 3. Results

### 3.1. Both Electroacupuncture and Moxibustion Generate Therapeutic Effects on UC Mice Model

The DSS-induced UC model mice generally were in poor conditions. Their hairs were sparse and had no luster; their excrement was soft and loose; the bloody excrement could be observed with naked eyes; and the DAI scores increased significantly. After the treatment of electroacupuncture and moxibustion, the vitality of mice and several symptoms such as mucopurulent stool, diarrhea, and other severe parameters have improved significantly (*P* < 0.05). Consequently, the DAI score has decreased significantly (*P* < 0.05) (Table 2).

**Figure 1:**
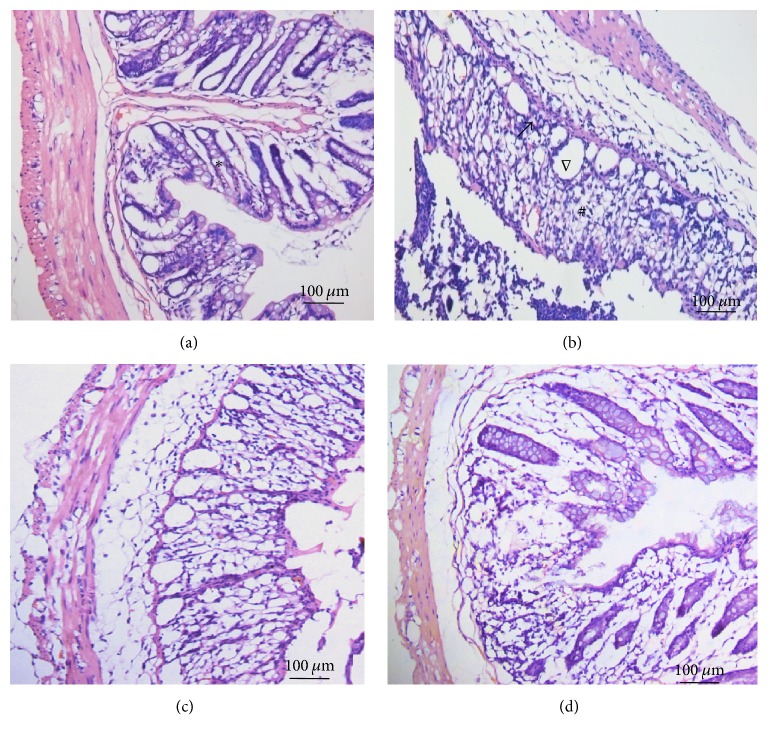
HE staining results of distal colonic tissues in different group. Normal mucosal epithelia, intestinal glands, and structure were seen in distal colonic tissues in the control group (a). Damaged mucosa, disordered glandular structure, and apparent edema in and underneath the mucosa were found in the DSS-induced model group mice (b). And the histopathological conditions have been greatly improved in acupuncture (c) and moxibustion group (d) (^*∗*^normal architecture and lack of cellular infiltrate; ^#^histological damage; ^∇^Globlet cell depletion*; magnification, 200x, and scale bar = 100 μm*).

Histopathology analysis showed that, for control group, there were normal mucosal epithelia, intestinal glands, and structure in distal colonic tissues. No signs of obvious ulceration or inflammatory cell infiltration were observed. In contrast, colonic tissues of rats from model group presented damaged mucosa, disordered glandular structure, and inflammatory cells infiltration. After electroacupuncture and moxibustion treatment, the histopathological conditions have been improved in different levels: the epithelial mucosa and glands are ordered and most of the infiltrating inflammatory cells have disappeared (Figure 1).

**Figure 2:**
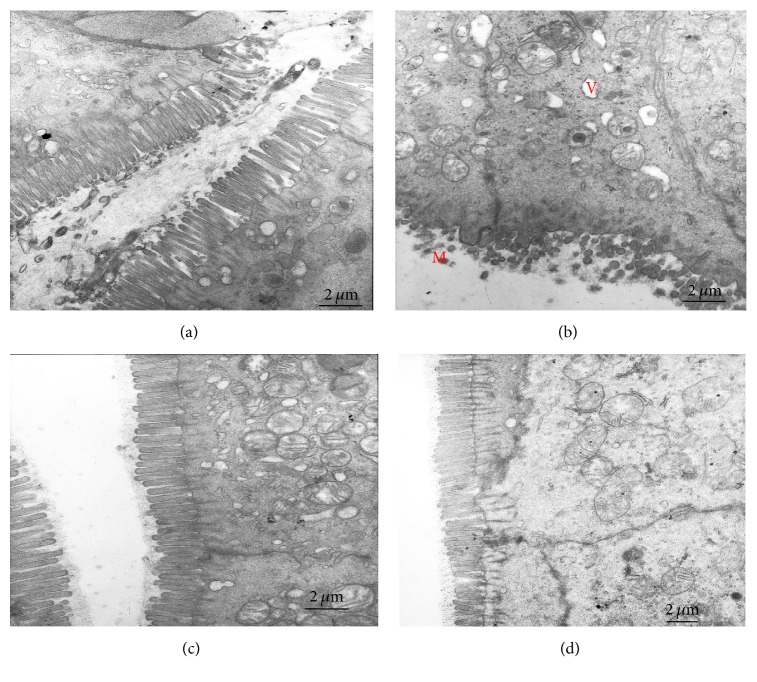
The ultrastructure inspection of the distal colon tissues by electron microscopy. Compared with the control group (a), the model group (b) has shown shortened loose microvilli with uneven length (M), large intraepithelial vacuoles (V), and unclear dissolved mucosal granules in the cytoplasm. After acupuncture (c) or moxibustion (d) treatment, the microvilli are in good order* (magnification, 10000x, and scale bar = 2 μm)*.

The ultrastructure inspection of the distal colon by electron microscopy indicates that much more shortened loose microvilli and large intraepithelial vacuoles were seen in the model group. However, more good order microvilli, normal mitochondria, and tight intracellular junctions are found in the electroacupuncture and moxibustion treated group (Figure 2).

**Figure 3:**
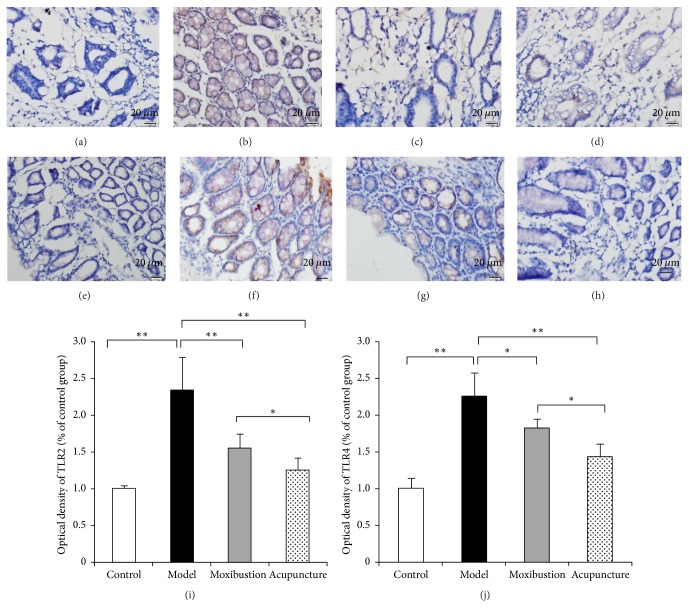
Toll-like receptor (TLR) 2 and TLR4 were detected in the colon by the immunohistochemical method. Compared with the control group (a, e), the intestinal TLR2 and TLR4 expression in normal in model group have increased (b, f). After acupuncture (c, g) or moxibustion (d, h) treatment, the expressions of TLR2 and TLR4 have been reduced. The average IOD was obtained by analyzing TLR2 (i) and TLR4 (j) immunohistochemistry staining in five random fields of each slide. Compared with electroacupuncture group, the OD level of TLR2 and TLR4 in moxibustion group is lower. ^*∗*^*P* < 0.05; ^*∗∗*^*P* < 0.01* (magnification, 200x, and scale bar = 100 μm).*

**Figure 4:**
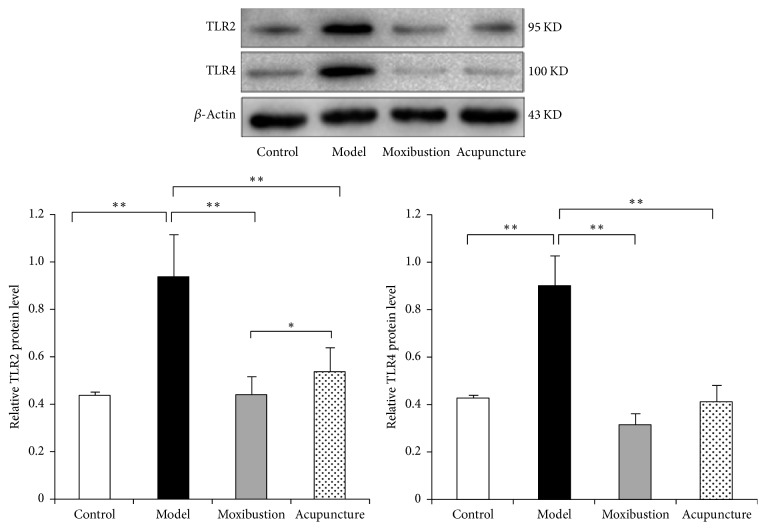
The protein expressions of TLR2 and TLR4 in the distal colon tissues. The results showed that the TLR2 and TLR4 protein expressions of the distal colon in model group were significantly enhanced, while, after acupuncture and moxibustion treatments, these two proteins were significantly reduced (*P* < 0.01). In addition, the level of TLR2 in moxibustion group is lower than that in the electroacupuncture group. ^*∗*^*P* < 0.05; ^*∗∗*^*P* < 0.01.

The immunohistochemical result has shown that in the model group the expressions of TLR2 and TLR4 were facilitated. The immunohistochemical marks which indicated the TLR2 protein and TLR4 protein in the model group obviously have increased as compared to that of the control group. Moreover, the marks have faded after either electroacupuncture or moxibustion treatment, which indicates that in both electroacupuncture and moxibustion groups their expressions have been reduced (Figure 3). Semiquantitative analysis showed that the relative optical density (OD) of TLR2 and TLR4 in the model group was much higher (compared with control group, *P* < 0.01). However, after acupuncture and moxibustion treatment, the relative OD values are both decreased (compared with model group, *P* < 0.01). As to the two treatment groups, moxibustion group has a relative lower level and is more close to the control group level (compared with electroacupuncture group, *P* < 0.05). Moreover, the results of western blot analysis (Figure 4) have also shown that the TLR2 and TLR4 protein expressions of the distal colon in model group are significantly enhanced, while, after electroacupuncture and moxibustion treatment, these two proteins have been significantly reduced (*P* < 0.01). In addition, the protein expression of TLR2 in moxibustion group is less than that in electroacupuncture group (*P* < 0.05), which indicated the effect of moxibustion was a little bit better than electroacupuncture.

### 3.2. Electroacupuncture and Moxibustion Enhance the Balance of Treg/Th17 Axis

**Figure 5:**
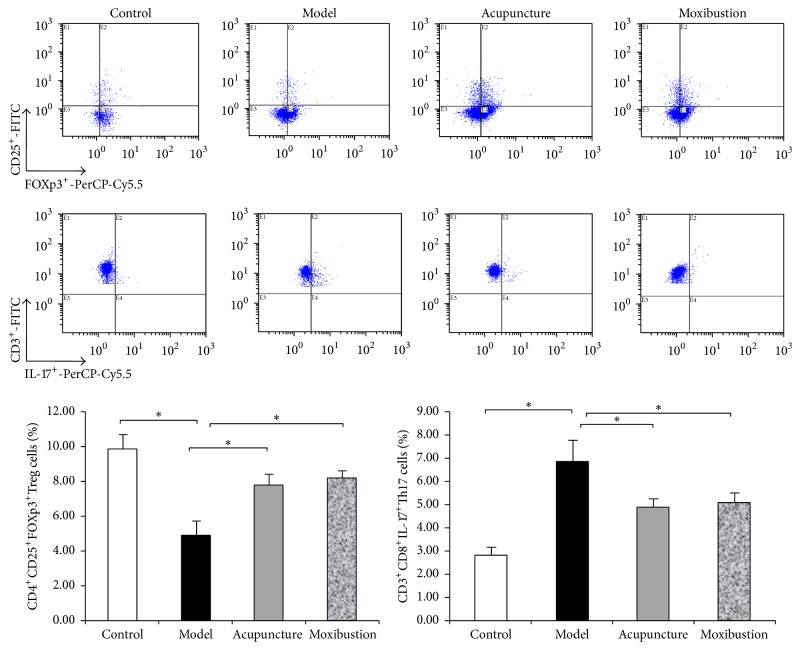
Flow cytometric pictures and the percentage of CD4^+^CD25^+^Foxp3^+^Treg cells and CD3^+^CD8^+^IL-17^+^Th17 cells among CD4^+^T cells in each group. Lymphocytes from spleen of each group were stained with labeled as anti-mice antibodies as in the described methods. Treg cells and Th17 were gated and shown in the right-upper quadrant. Compared with control group, the percentage of Treg (CD4^+^CD25^+^Foxp3^+^Treg/CD4+ T cells) in model group decreased (*P* < 0.05). Treg cells content increased after acupuncture or moxibustion treatment (*P* < 0.05). On the contrary, the percentage of Th17 cells significantly increased (*P* < 0.05) in UC model mice while decreasing by both acupuncture and moxibustion. ^*∗*^*P* < 0.05.

Compared with the control group, CD4+CD25+Foxp3+Treg cells in spleen are significantly decreased, and CD3+CD8+IL-17+Th17 cells are also significantly increased (*P* < 0.05) in the UC model mice. After electroacupuncture and moxibustion treatment, CD4+CD25+Foxp3+Treg cells are significantly increased, and CD3+CD8+IL-17+Th17 cells are significantly decreased (*P* < 0.05). However, there is no obvious difference between electroacupuncture group and moxibustion group (*P* > 0.05) (Figure 5).

### 3.3. Electroacupuncture and Moxibustion Improve Key Immune Factors That Are Regulated by Treg/Th17 Axis

**Figure 6:**
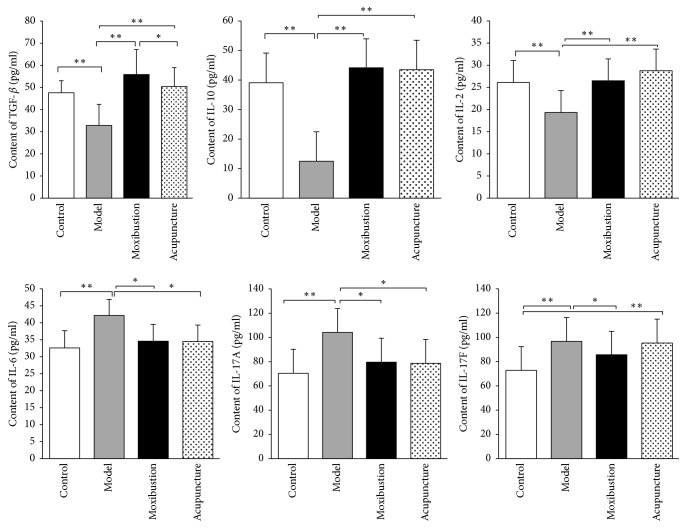
Quantification of the key immune factors by ELISA test: TNF-*β*, IL-10, and IL-2; IL-6, IL-17A, and IL-17F. The results showed that the levels of anti-inflammatory factors including TGF-*β*, IL-10, and IL-2 in the UC model group significantly decreased (Figure 6, upper panel; as compared with control group *P* < 0.01). On the contrary, the levels of proinflammatory factors including IL-6, IL-17A, and IL-17F significantly increased after modeling (Figure 6, lower panel; as compared with control group *P* < 0.01). After acupuncture and moxibustion treatments, the levels of TGF-*β*, IL-10, and IL-2 were significantly increased (Figure 6, upper panel) and the levels of IL-6, IL-17A, and IL-17F were significantly reduced (lower panel). In addition, there is no obvious difference between them in all these inflammation factors. ^*∗*^*P* < 0.05; ^*∗∗*^*P* < 0.01.

There are also significant changes in the quantity of immune factors in the model group. The levels of anti-inflammatory factors including TGF-*β*, IL-10, and IL-2 have significantly decreased as compared to that of the control group (*P* < 0.01). On the contrary, the levels of proinflammatory factors including IL-6, IL-17A, and IL-17F have significantly increased after modeling. Moreover, the changes in these immune factors have greatly reverted after either electroacupuncture or moxibustion treatment. The quantification analysis of these immune factors indicates that the quantities of TGF-*β*, IL-10, and IL-2 have significantly increased in electroacupuncture group (*P* < 0.01) and moxibustion group (*P* < 0.01). Similarly, the levels of IL-6, IL-17A, and IL-17F have significantly been reduced after treatment (*P* < 0.01 for IL-6 and *P* < 0.05 for IL-17A and IL-17F). However, there is no obvious difference between electroacupuncture group and moxibustion group (*P* > 0.05) (Figure 6).

### 3.4. Electroacupuncture and Moxibustion Might Rebalance the Treg/Th17 Axis through Regulating the Activity of Upper Stream Proteins

**Figure 7:**
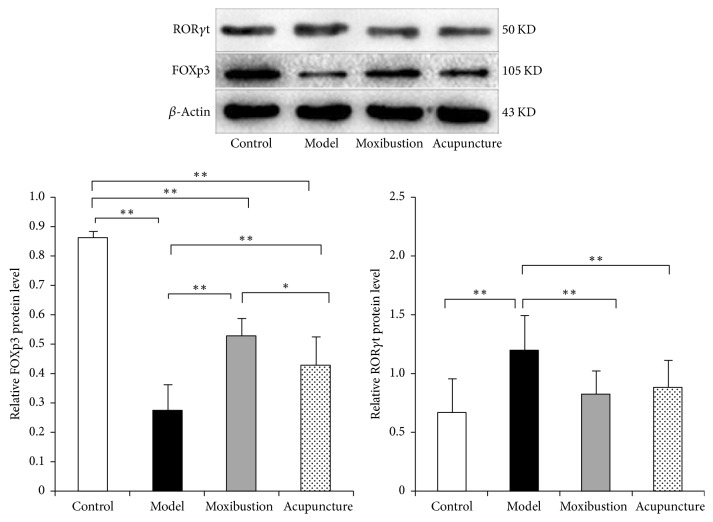
The protein expressions of ROR*γ*t and FOXP3 in the distal colon tissues. Compared with controls, the ROR*γ*t expression was significantly increased and FOXp3^+^ expression was significantly decreased in the UC model group (*P* < 0.01). However, after acupuncture or moxibustion treatment, ROR*γ*t expression was obviously decreased (*P* < 0.01) and FOXP3 expression was increased (*P* < 0.01). In addition, moxibustion increased higher FOXP3 than electroacupuncture. ^*∗*^*P* < 0.05; ^*∗∗*^*P* < 0.01.

Results of western blot analysis (Figure 7) have shown that in model group the ROR*γ*t protein expression of the distal colon is enhanced, while that of the FOXP3 protein is weakened as compared with that of the control group. Electroacupuncture and moxibustion treatments have significantly improved the expressions of these two important proteins that were in the upper stream of the Treg/Th17 axis. Compared with model group, the expression of the ROR*γ*t protein is significantly reduced in electroacupuncture and moxibustion groups (*P* < 0.01). As to the FOXP3 protein, its expression is significantly upregulated in electroacupuncture and moxibustion groups when compared with that of the model group (*P* < 0.01) (Figure 7). Compared with electroacupuncture group, the protein expression of FOXP3 in moxibustion group is higher than electroacupuncture group.

## 4. Discussions

The current study intends primarily to assess the effects of electroacupuncture and moxibustion in managing UC symptoms. Results have shown that both of these therapeutics can significantly improve the symptoms of UC as indicated by the measurement of DAI which is adopted in most clinical [25] and animal researches [26]. Further comparison has also indicated that both electroacupuncture and moxibustion may regulate the intestinal immune response mediated by rebalancing the Treg/Th17 axis [27]. Actually, this is the key to both the development of UC and its successful treatment. This study has also provided some evidences that moxibustion may be better than electroacupuncture in alleviating UC based on the protein expression of TLR2 and TLR4. Also, moxibustion has better effect in increasing the level of FOXP3 protein. However, whether this effect is related to Treg/Th17 balance has not been clear yet because the cytokines and the ROR*γ*t protein expression are not so convincing. These results provided new clues for investigating and discussing the different mechanism of acupuncture and moxibustion in treating UC. More detailed discussions of the aforementioned viewpoints are described as follows.

### 4.1. Treg/Th17 Balance Has a Crucial Impact on UC

It is well known that there are many factors involved in the pathogenesis of UC. One of the main causes is believed to be the involvement of a dysregulated immune response to some unknown stimulus [28]. Th17/Treg balance may play a crucial role in this inflammatory and immunological process of UC [8]. Due to the imbalance of Th17 and Treg, serum concentrations of Th17-related cytokines (IL-6, IL-17A, and IL-17F) will increase and Treg-related cytokines (IL-10, IL-2, and TGF-*β*) will decrease accordingly [29]. In addition, the cytokines can exert synergistic effects in stimulating the injury of colonic epithelial cells, which will then contribute to the formation of local tissue inflammation. Recovery of the Th17/Treg immune balance may imply new therapeutic targets in UC management [27]. FOXP3 is a master regulator for the development and function of Treg cells. As a transcription factor of Treg cells, it is crucial for the induction and maintenance of the unique immune-suppressive properties of Treg cells [30]. Mutations within the FOXP3 gene or deletion of FOXP3 in transgenic mouse models can result in the development of fatal autoimmunity. ROR*γ*t innate lymphoid cells play a fundamental role in the development of lymphoid tissues, as well as in homeostasis and defense of mucosal tissues [31]. These cells produce key cytokines, IL-22 and IL-17A, for the activation of epithelial defenses and the recruitment of polymorphonuclear phagocytes. In the absence of ROR*γ*t innate lymphoid cells, the early defense to infection and resistance to injury are compromised.

A number of studies have shown that inflammation cytokines or inflammatory protein was responsible for UC inflammation [32, 33]. Besides, many studies showed that, in the absence of TLR stimuli, Tregs dampened B cell proliferation, plasma cell formation, and Ig production [34]. What is more, TLR2 stimulation drives human naive and effectors regulatory T cells into a Th17-like phenotype with reduced suppressive function and regulates the balance between regulatory T cell and Th17 function [35, 36], thereby promoting disease activity and progression. In this study, we revealed that the trends of serum IL-10, IL-2, IL-6, IL-17A, and IL-17F in DSS-induced UC mice coincide with previous reports. In addition, the levels of protein expressions of TLR2 and TLR4 in intestinal tissues get the similar results too. Thus, the aforementioned results have provided further and relative comprehensive evidences for the importance of evaluating the levels of Th17/Treg axis in UC development. So far, our results have demonstrated that the balance of peripheral Treg/Th17 is disturbed in UC mice, and the Treg/Th17 imbalance might act synergistically with microinflammation on the development of UC. Concurrently, our data have also shown a reciprocal relationship between Treg and Th17 cells numbers, as well as between Treg and Th17-related cytokines. These results indicate that the Treg/Th17 immune axis may be involved in the mechanism of UC progression and the functional imbalance of Treg/Th17 may have a potential impact on it.

### 4.2. Electroacupuncture and Moxibustion Can Ameliorate the Balance of Treg/Th17 in UC Mice

Currently, patients with inflammatory bowel disease (IBD) or irritable bowel syndrome (IBS) have either very few effective treatments or some modestly effective treatments which are not entirely free from risks. Thus, seeking safe and effective treatments for these diseases has been imperative. Moxibustion and electroacupuncture, although their effects should be further improved by rigorously designed clinical trials, have gained much attention as alternative and complementary therapeutic interventions due to their relatively low clinical side effects as compared to drug therapy or surgical procedures [37–39]. When moxibustion is added as an adjunct to other treatments, it significantly reduces symptoms of IBD or IBS relative to other treatments alone. Joos et al. [38] have suggested that acupuncture offers an additional therapeutic benefit in patients with mild to moderately active UC.

Most researches have focused on whether moxibustion and acupuncture are effective, and very few have investigated the mechanistic connection between moxibustion and acupuncture therapy. Wu et al. [40] have suggested that moxibustion and acupuncture inhibited expression of inflammatory cytokines by observing IL-1*β* and IL-6 mRNA expression in the spleen and colonic mucosa of UC rats. In Han's study [26], moxibustion treatment improves disease activity, repairs damaged colonic mucosa, and suppresses secretion of serum IL-8 while it activates that of IL-10, inhibits activation of NF-*κ*B p65, and decreases expression of TLR-9 in UC rats [26]. In our study, results indicate that both electroacupuncture and moxibustion treatment can improve disease activity, repair damaged colonic mucosa, decrease the protein expression of TLR2 and TLR4, increase the content of CD4+CD25+Foxp3+ Treg cells, and decrease the level of CD3+CD8+IL-17+Th17 cells. In addition, after electroacupuncture and moxibustion treatment, the levels of TGF-*β*, IL-10, and IL-2 have significantly increased and the levels of IL-6, IL-17A, and IL-17F have significantly reduced, indicating electroacupuncture and moxibustion can inhibit the inflammatory cells by suppressing secretion of proinflammatory cytokine and activate secretion of anti-inflammatory cytokine. To further verify the proposed idea that the effects of electroacupuncture and moxibustion therapy in UC mice are probably related to Th17/Treg immune axis, we have detected the protein expressions of FOXP3 and ROR*γ*t. The results have shown that the ROR*γ*t expression has significantly increased and FOXp3 expression has significantly decreased in the UC model mice, but after either electroacupuncture or moxibustion treatment, ROR*γ*t expression has obviously decreased and FOXP3 expression has increased almost to the normal level, indicating that both electroacupuncture and moxibustion therapies can maintain the equilibrium of Th17/Treg immune axis in UC mice.

### 4.3. To Some Extent, Moxibustion Has Better Efficacy Than Electroacupuncture in Treating UC, but Its Relationship to Treg/Th17 Axis Is Not Clear Yet

There is an increasing number of clinical studies of electroacupuncture and moxibustion treatments for IBD [41, 42] and IBS [43], and the existing investigations have partially demonstrated that electroacupuncture and moxibustion can effectively control bowel inflammation by providing multitargeted regulation of the body's physiological balance. However, it is imperative to know if there is actually any difference between these two treatments in activating acupoints and treating UC. A system review [42] has indicated that, among the 43 included RCT studies, acupuncture and moxibustion therapies were alternatively used in 17 studies [44–60]; moxibustion treatment was used as the main intervention in 12 studies [44–53, 58, 59], while acupuncture alone was used in one study [60]; combinations of two types of acupuncture and moxibustion treatments were used in 10 studies [38, 61–69]. Other studies combined acupuncture, moxibustion, and traditional Chinese herb or cupping or other methods [70–75]. According to this review, moxibustion has been more widely used in treating UC, but no study has explicitly indicated the difference and the reason why acupuncture is not widely used as moxibustion. In the current study, we have found that, at least to some extent, moxibustion demonstrates better efficacy than acupuncture. For example, in ameliorating the level of TLR2 and TLR4 (we mentioned above that they are closely related to intestinal inflammation), the effect of moxibustion is better than that of electroacupuncture (*P* < 0.05). In addition, moxibustion can obviously improve the protein level of FOXP3 (*P* < 0.05). However, based on our results, whether the better curative effect of moxibustion is related to Treg/Th17 balance has not been clear yet. For one thing, the percentage of CD4+CD25+Foxp3+Treg cells and CD3+CD8+IL-17+Th17 cells among CD4+T cells in the two treatment groups has no obvious difference (*P* > 0.05). For another, the cytokines of IL-10, IL-2, IL-6, IL-17A, and IL-17F and the protein expression of ROR*γ*t have no difference either. Further study should be carried out to prove that.

In summary, this study proved that both electroacupuncture and moxibustion can ameliorate UC and their effects are partially from the way of improving the balance of Th17/Treg. In addition, our study indicated whether moxibustion has better efficacy than electroacupuncture needs further study.

## Figures and Tables

**Table 1:** Ulcerative colitis disease activity index.

Body weight loss (%)	Characteristics of feces	Fecal occult blood/gross fecal blood	Scoring
0	Normal	Normal	0
1–5	Loose	Fecal occult blood	1
6–10	Loose	Fecal occult blood	2
11–15	Watery feces	Naked eye bloody feces	3
>16	Watery feces	Naked eye bloody feces	4

**Table 2:** Comparison of DAI in each group (means ± SD).

Group	Sample	After modeling	After treatment
Control group	—	—	—
Model group	12	2.67 ± 0.49	2.61 ± 0.51
EA-group	12	2.64 ± 0.52	1.11 ± 0.59^*∗*^
Moxibustion group	12	2.61 ± 0.44	1.05 ± 0.49^*∗*^

*Note*. ^*∗*^Compared with model group (*P* < 0.05).
